# Supplementary material for: Disease-relevant upregulation of P2Y1 receptor in astrocytes enhances neuronal excitability via IGFBP2
Source: Nat Commun. 2024 Aug 8;15:6525. doi: 10.1038/s41467-024-50190-7 (PMC11310333; doi:10.1038/s41467-024-50190-7)
Supplement: Supplementary file 1 — Supplementary Information [file 41467_2024_50190_MOESM1_ESM.pdf]

Supplementary Information

Disease-relevant upregulation of $P2Y_1$ receptor in astrocytes enhances neuronal excitability via IGFBP2

Eiji Shigetomi[1,2*,Ψ], Hideaki Suzuki[1,2,*], Yukiho J. Hirayama[1], Fumikazu Sano[1,2,3], Yuki Nagai[1,2], Kohei Yoshihara[4], Keisuke Koga[4,5], Toru Tateoka[6], Hideyuki Yoshioka[6], Youichi Shinozaki[1,2], Hiroyuki Kinouchi[6], Kenji F. Tanaka[7], Haruhiko Bito[8], Makoto Tsuda[4,9] & Schuichi Koizumi[1,2,Ψ]

[1]Department of Neuropharmacology, Interdisciplinary Graduate School of Medicine, University of Yamanashi, Yamanashi 409-3898, Japan.
[2]Yamanashi GLIA center, University of Yamanashi, Yamanashi 409-3898, Japan.
[3]Department of Pediatrics, Faculty of Medicine, University of Yamanashi, Yamanashi 409-3898, Japan.
[4]Department of Molecular and System Pharmacology, Graduate School of Pharmaceutical Sciences, Kyushu University, Fukuoka 812-8582, Japan.
[5]Department of Neurophysiology, Hyogo College of Medicine, Hyogo 663-8501, Japan.
[6]Department of Neurosurgery, Interdisciplinary Graduate School of Medicine, University of Yamanashi, Yamanashi 409-3898, Japan.
[7]Division of Brain Sciences, Institute for Advanced Medical Research, Keio University School of Medicine, Tokyo 160-8582, Japan.
[8]Department of Neurochemistry, Graduate School of Medicine, The University of Tokyo, Tokyo 113-0033, Japan.
[9]Department of Life Innovation, Graduate School of Pharmaceutical Sciences, Kyushu University, Fukuoka 812-8582, Japan.

[Ψ]Corresponding author. Eiji Shigetomi, eshigetomi@yamanashi.ac.jp; Schuichi Koizumi, skoizumi@yamanashi.ac.jp

This file includes:

Supplementary Figures 1 to 9

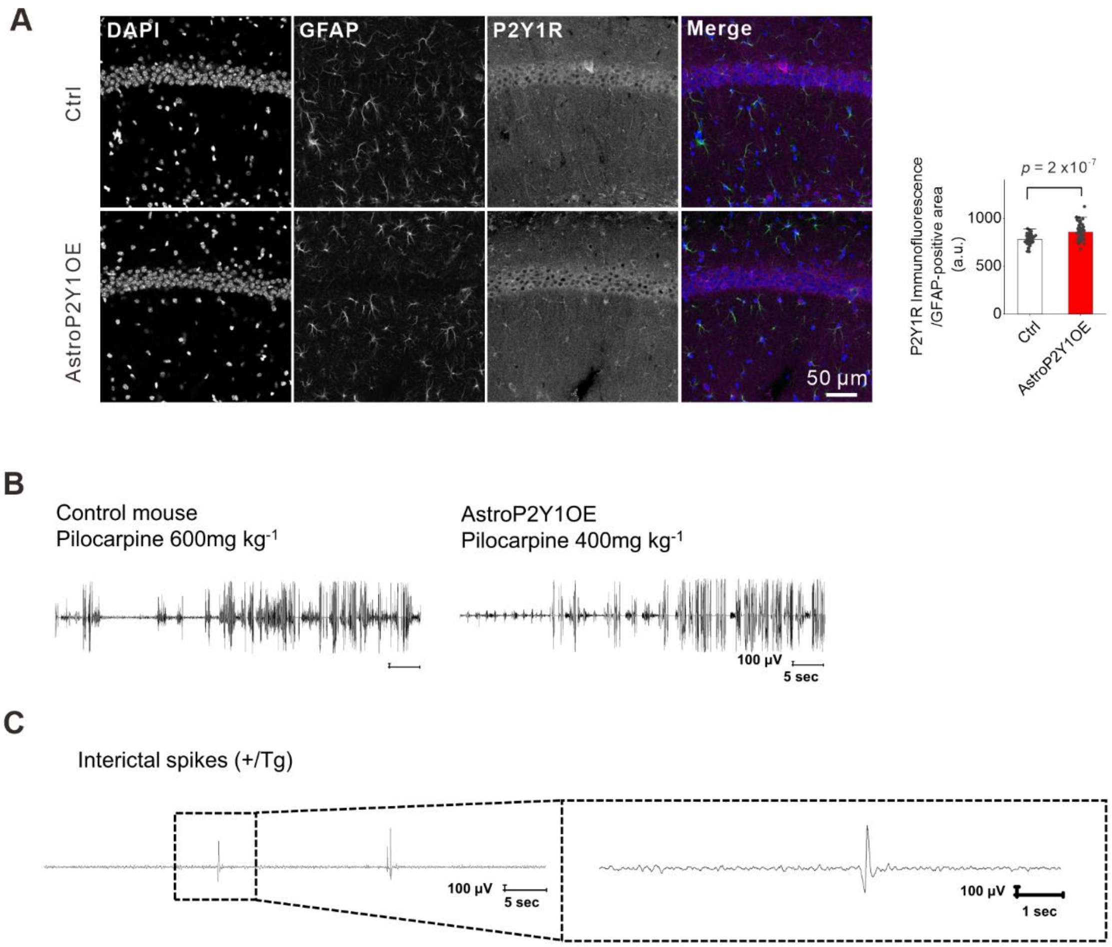

Supplementary Figure 1. Additional traces for EEG recordings.

A. Immunohistochemistry of anti-P2Y1R in hippocampal CA1 region in AstroP2Y1OE and control mice. P2Y1R immunofluorescence signals in GFAP-positive area were higher in AstroP2Y1OE. Control, n=54 ROIs, n=3 slices, 3 mice; AstroP2Y1OE, n=59 ROIs, n=3 slices, 3 mice (two-sided two-sample t-test). Data are presented as mean ± s.d. B. Pilocarpine-induced epileptic spike discharges. C. Expanded traces for interictal spikes in AstroP2Y1OE mice. Source data are provided as a Source Data file.

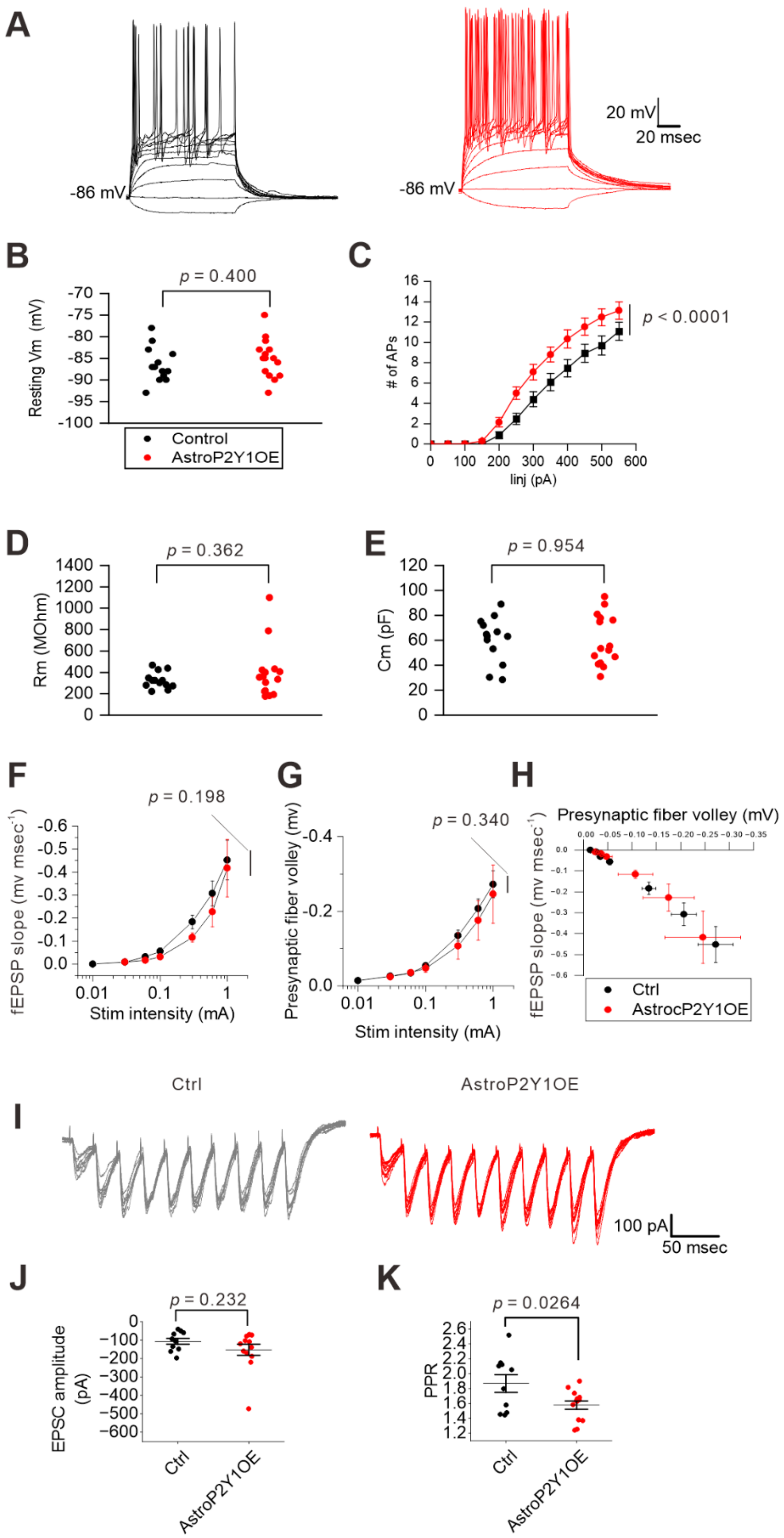
A
20 mV
20 msec
-86 mV
-86 mV
B
p = 0.400
Resting Vm (mV)
Control
AstroP2Y1OE
C
p < 0.0001
# of APs
Iinj (pA)
D
p = 0.362
Rm (MOhm)
E
p = 0.954
Cm (pF)
F
p = 0.198
fEPSP slope (mv msec-1)
Stim intensity (mA)
G
p = 0.340
Presynaptic fiber volley (mv)
Stim intensity (mA)
H
Presynaptic fiber volley (mV)
fEPSP slope (mv msec-1)
Ctrl
AstrocP2Y1OE
Ctrl
AstroP2Y1OE
I
100 pA
50 msec
J
p = 0.232
EPSC amplitude (pA)
Ctrl
AstroP2Y1OE
K
p = 0.0264
PPR
Ctrl
AstroP2Y1OE

Supplementary Figure 2. Synaptic and membrane properties of neurons in AstroP2Y1OE and control mice.

A. Representative traces of membrane potentials of granule neurons in the dentate gyrus, induced by current injection steps. B-H. Black circles, control; red circles, AstroP2Y1OE. B. Summary of resting membrane potentials (two-sided two-sample t-test). C. Summary of the number of action potentials induced by the current injection steps. Neurons in AstroP2Y1OE mice had more action potentials. Control, n=13 neurons from 13 slices, 5 mice; AstroP2Y1OE, n=12 neurons from 12 slices, 4 mice (two-way repeated measures ANOVA and Tukey's test). Data are presented as mean ± s.e.m. D, E. Summary of input membrane resistance (D) and membrane capacitance (F) (two-sided two-sample t-test). F, G. Field excitatory postsynaptic potential slopes (F) and presynaptic fiber volley amplitudes (G) were recorded from the stratum radiatum in the CA1 region of hippocampal slices. Control, n=9 slices, 4 mice; AstroP2Y1OE, n=6 slice, 4 mice (two-way repeated measures ANOVA and Tukey's test). Data are presented as mean ± s.e.m. H. Relationship between the field excitatory postsynaptic potential slopes and the amplitudes of presynaptic fiber volleys. Data are presented as mean ± s.e.m. I. Membrane currents recorded from CA1 pyramidal neurons in the presence of picrotoxin (100 μM). The Schaffer collaterals were stimulated at 40Hz, 10 times, 0.06 mA. >90% of evoked EPSC amplitude was blocked by CNQX (10 μM, data not shown). J. Summary of the first EPSC amplitude. Control, n=10 cells from 10 slices, 8 mice; AstroP2Y1OE, n=13 cells from 13 slices, 7 mice (two-sided Mann-Whitney U test). Data are presented as mean ± s.e.m. K. Summary of the paired-pulse ratio (PPR) of the first and the second EPSCs. PPR was lower in AstroP2Y1OE. Control, n=10 cells from 10 slices, 8 mice; AstroP2Y1OE, n=13 cells from 13 slices, 7 mice (two-sided two-sample t-test). Data are presented as mean ± s.e.m. Source data are provided as a Source Data file.

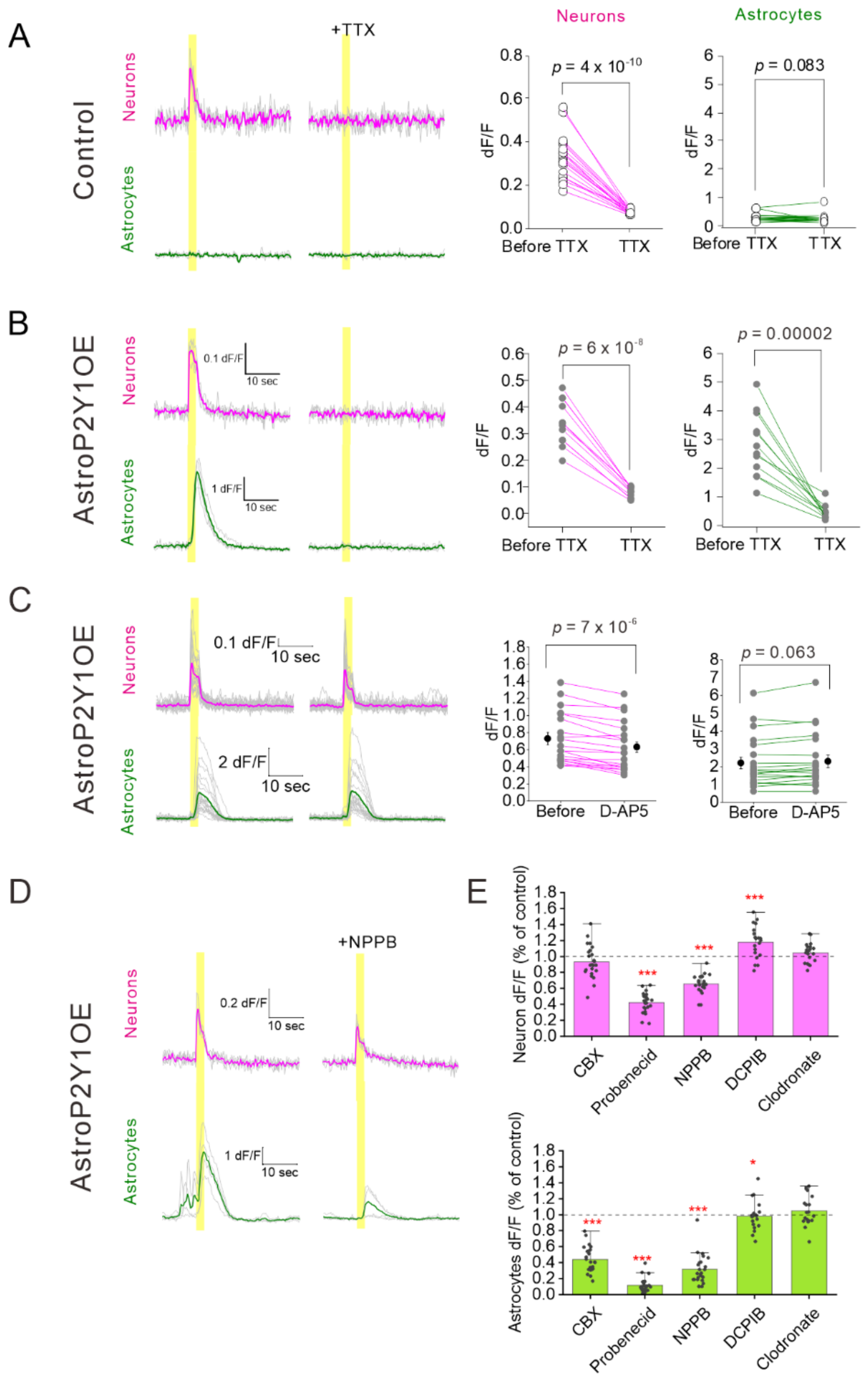

Supplementary Figure 3. Pharmacological analysis of neuronal and astrocytic Ca^{2+} signals evoked by EFS.

EFS-evoked Ca^{2+} signals in neurons and astrocytes. EFS (40Hz, 100 times, 0.06 mA) was delivered at the duration indicated by yellow boxes. A. Sample traces and summary bar graphs

for TTX (1 μM) treatment in control mice. n=20 ROIs from 5 slices, 3 mice (two-sided paired t-test). B. Sample traces and summary bar graphs for TTX treatment in AstroP2Y1OE mice. n=12 ROIs from 3 slices, 3 mice (two-sided paired t-test). TTX abolished both neuronal and astrocytic Ca^{2+} signals. C. Sample traces and summary bar graphs for D-AP5 (50 μM) treatment in AstroP2Y1OE mice. n=20 ROIs from 6 slices of 3 mice (two-sided paired t-test). Data are presented as mean ± s.e.m. D. Sample traces for NPPB (100 μM) treatment in slices from AstroP2Y1OE mice. Representative traces of dual-color Ca^{2+} imaging before and during NPPB. D. Effects of the pharmacological inhibition of ATP release pathways on both neuronal and astrocytic Ca^{2+} signals. CBX (100 μM), n=24 ROIs from 6 slices, 3 mice; probenecid (5 mM), n=24 ROIs from 6 slices, 4 mice; NPPB (100 μM), n=24 ROIs from 6 slices, 3 mice; DCPIB (20 μM), n=20 ROIs from 5 slices, 3 mice; clodronate (100 μM), n=20 ROIs from 5 slices, 4 mice. ***$p < 0.001$, *$p < 0.05$ (two-sided Mann–Whitney U test). Neuron+probenecid, $p = 2 \times 10^{-10}$; Neuron+NPPB, $p = 2 \times 10^{-10}$; Neuron+DCPIB, $p = 5 \times 10^{-4}$; Astrocyte+CBX, $p = 2 \times 10^{-10}$; Astrocyte+probenecid, $p = 2 \times 10^{-10}$; Astrocyte+NPPB, $p = 2 \times 10^{-10}$; Astrocyte+CBX, $p = 0.02152$. Data are presented as mean ± s.d. Source data are provided as a Source Data file.

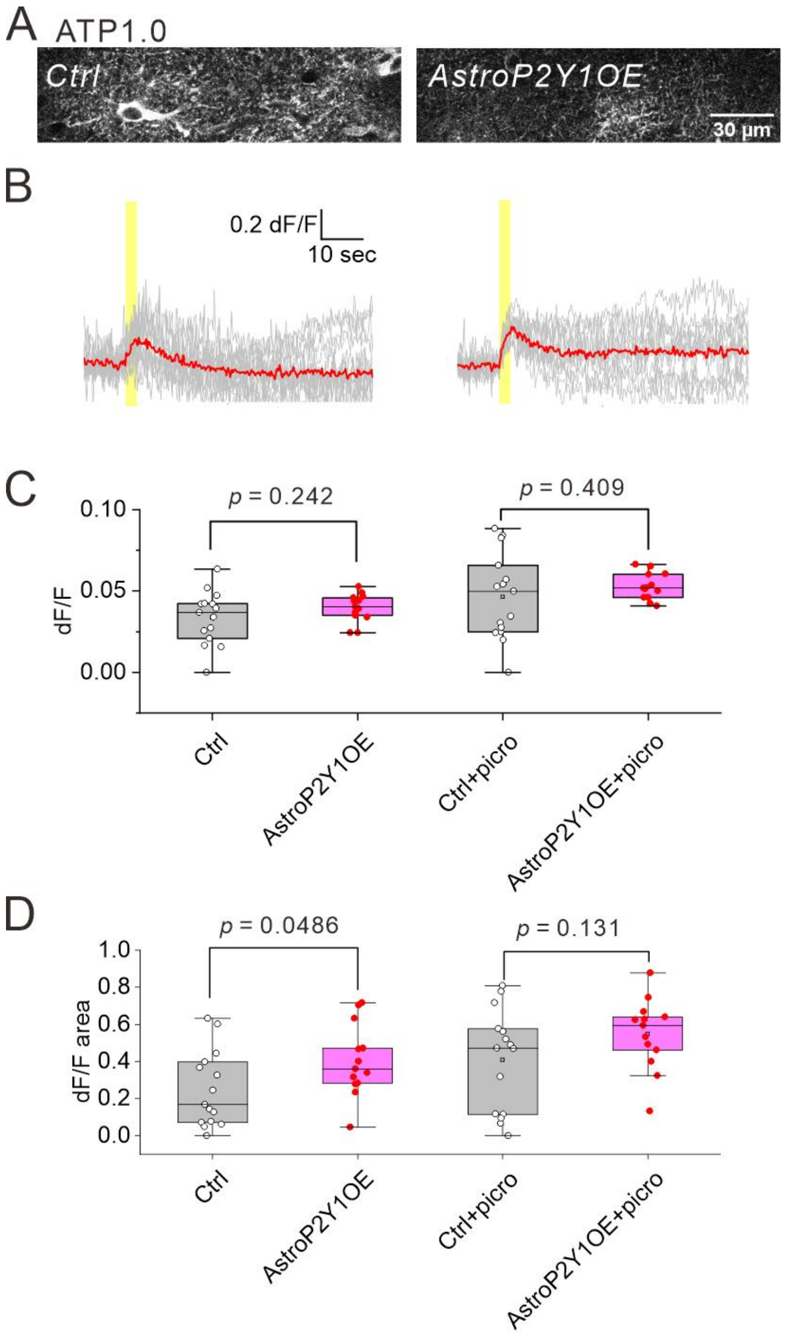

upplementary Figure 4. Extracellular ATP imaging by $GRAB_{ATP1.0}$ sensor in astrocytes.

A. Sample images of $GRAB_{ATP1.0}$-expressing astrocytes. B. Sample traces of ATP responses evoked by EFS (40Hz, 100 times, 0.06 mA). C, D. Summary of EFS-evoked ATP responses at the peak and area under the curve (D). Control, n=15 ROIs from 5 slices, 3 mice; AstroP2Y1OE, n=15 ROIs from 6 slices, 3 mice (two-sided two-sample t-test). Box-plot elements are defined in the following way: center line, median; box limits, upper and lower quartiles; whiskers, 1.5x interquartile range; square, mean. Source data are provided as a Source Data file.

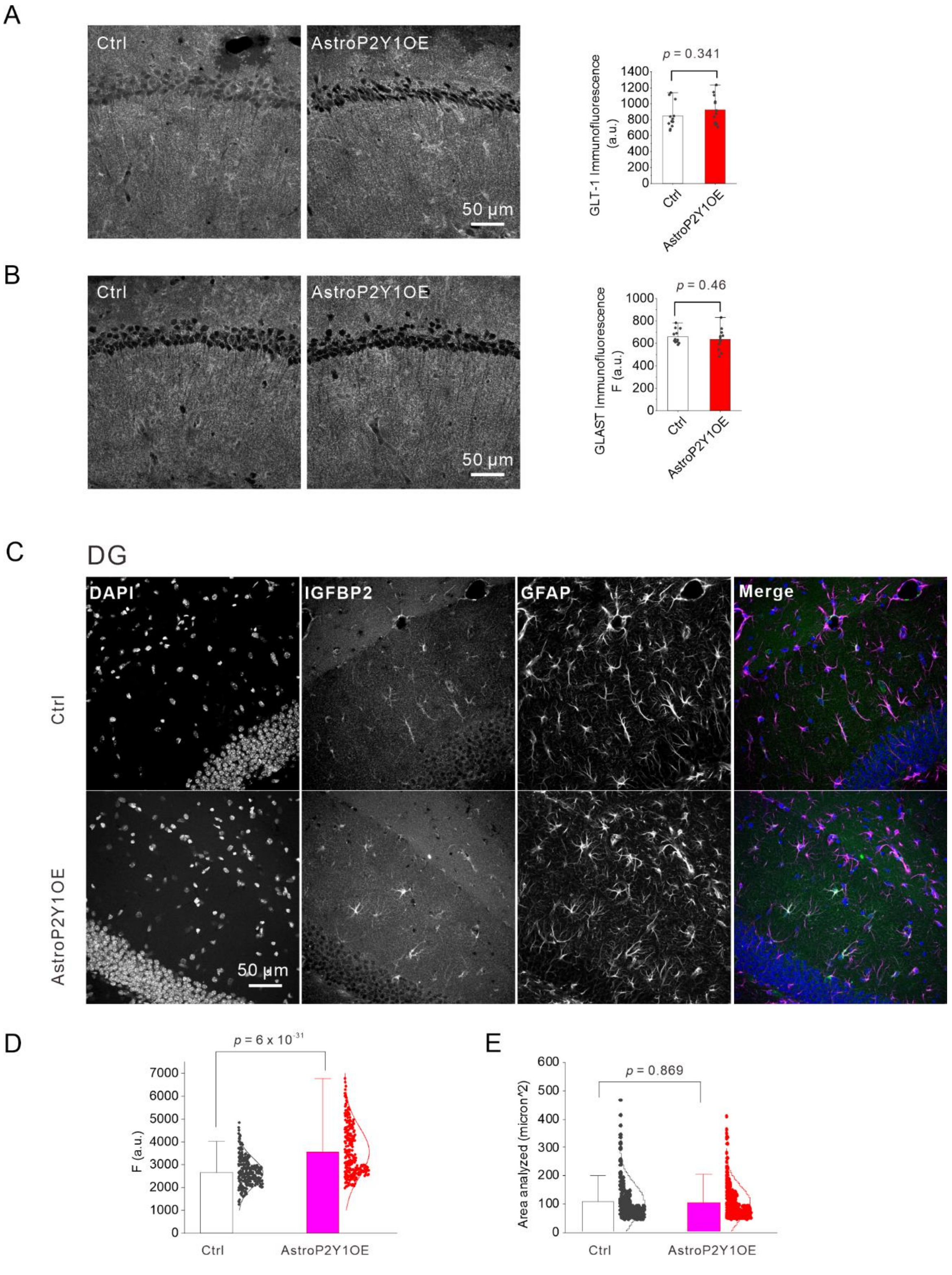
A
Ctrl
AstroP2Y1OE
50 µm
p = 0.341
GLT-1 Immunofluorescence (a.u.)
1400
1200
1000
800
600
400
200
0
Ctrl
AstroP2Y1OE
B
Ctrl
AstroP2Y1OE
50 µm
p = 0.46
GLAST Immunofluorescence F (a.u.)
1000
800
600
400
200
0
Ctrl
AstroP2Y1OE
C
DG
DAPI
IGFBP2
GFAP
Merge
Ctrl
AstroP2Y1OE
50 µm
D
p = 6 x 10^-31
F (a.u.)
7000
6000
5000
4000
3000
2000
1000
0
Ctrl
AstroP2Y1OE
E
p = 0.869
Area analyzed (micron^2)
600
500
400
300
200
100
0
Ctrl
AstroP2Y1OE

Supplementary Figure 5. Immunohistochemistry of IGFBP2 in the dentate gyrus of AstroP2Y1OE and control mice.

A. Sample images and summary of GLT-1 immunofluorescence signals. n=12 slices from 3 mice for each group (two-sided Mann-Whitney U test). Data are presented as mean ± s.d. B. Sample images and summary of GLAST immunofluorescence signals. n=12 slices from 3 mice for each group (two-sided Mann-Whitney U test). Data are presented as mean ± s.d. C. Sample images of immunohistochemical analysis of IGFBP2 expression in the hippocampus. GFAP was used as an astrocytic marker. D, E. Summary of IGFBP2 immunofluorescence signals in GFAP-positive area (D) and GFAP-positive area (E). Control, n=285 ROIs from 12 FOVs, 3 mice; AstroP2Y1OE, n=388 ROIs from 12 FOVs, 3 mice (two-sided two-sample t-test). Compared with the CA1 region, there was a trend toward higher IGFBP2 expression in astrocytes in the dentate gyrus. Data are presented as mean ± s.d. Black and red circles indicate values from individual ROIs from control and AstroP2Y1OE slices, respectively. Source data are provided as a Source Data file.

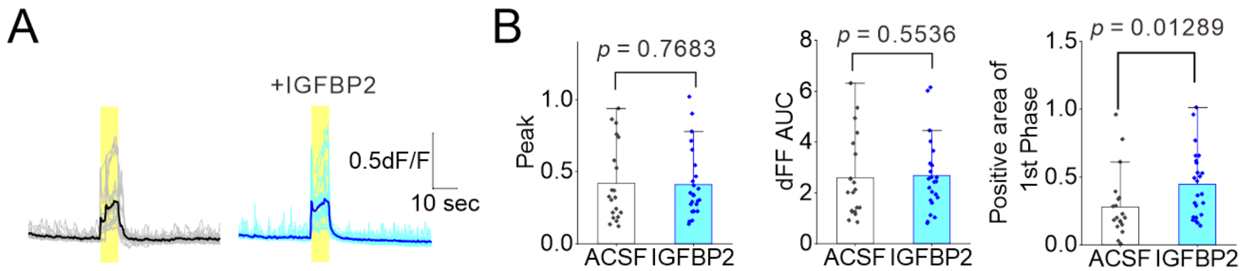

Supplementary Figure 6. Effects of IGFBP2 on dendritic Ca^{2+} signals.

A. Traces of dendritic Ca^{2+} signals. ROIs were set in the stratum radiatum of the images. B. Summary of the effects of IGFBP2 treatment (10 ng/mL for 1 hour at room temperature) on dendritic Ca^{2+} signals. ACSF (control), n=20 ROIs from 5 slices, 3 mice; IGFBP2, n=24 ROIs from 6 slices, 3 mice (two-sided Mann–Whitney U test). Data are presented as mean ± s.d. Source data are provided as a Source Data file.

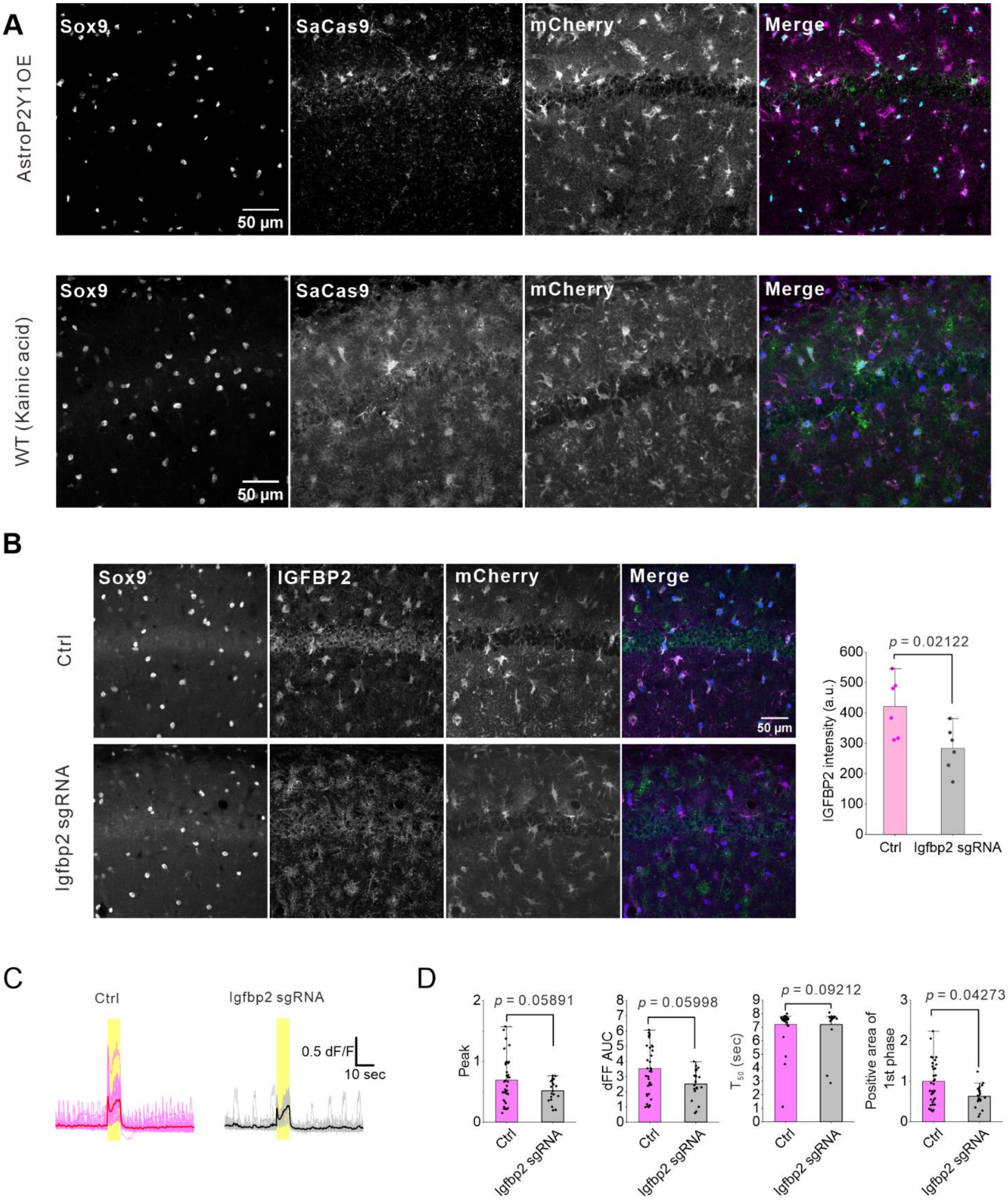

Supplementary Figure 7. Validation of IGFBP2 knockdown in astrocytes and effects of IGFBP2 knockdown on dendritic Ca^{2+} signals.

A. Immunohistochemistry of anti-SOX9, anti-HA (for SaCas9), and mCherry. Here, 62% ± 10% (n=4 fields of view from 2 mice) and 63% ± 6% (n=8 fields of view from 8 mice) of SOX9-

positive cells expressed SaCas9 and mCherry in P2Y1R transgenic and C57Bl6/j mice (kainic acid injection), respectively. B. IGFBP2 expression in astrocytes with or without sgRNA against Igfbp2 (two-sided two-sample t-test). IGFBP2 expression was significantly reduced, by approximately 30%. Data are presented as mean ± s.d. C. Effects of astrocytic knockdown of Igfbp2 on dendritic Ca^{2+} signals. Images used for the analysis were the same as those used in Figure 5f but the ROIs were set in the stratum radiatum. D. Summary of the dF/F peak, dF/F AUC, and T50 values. Control, n=36 cells from 9 slices, 4 mice; Igfbp2 gRNA, n=20 cells from 5 slices, 3 mice (two-sided two-sample t-test for dF/F peak, two-sided Mann–Whitney U test for dF/F AUC, T_{50}, and Positive area of the first phase). Although it was not significant, there was a trend toward decreased Ca^{2+} signals. Data are presented as mean ± s.d. Source data are provided as a Source Data file.

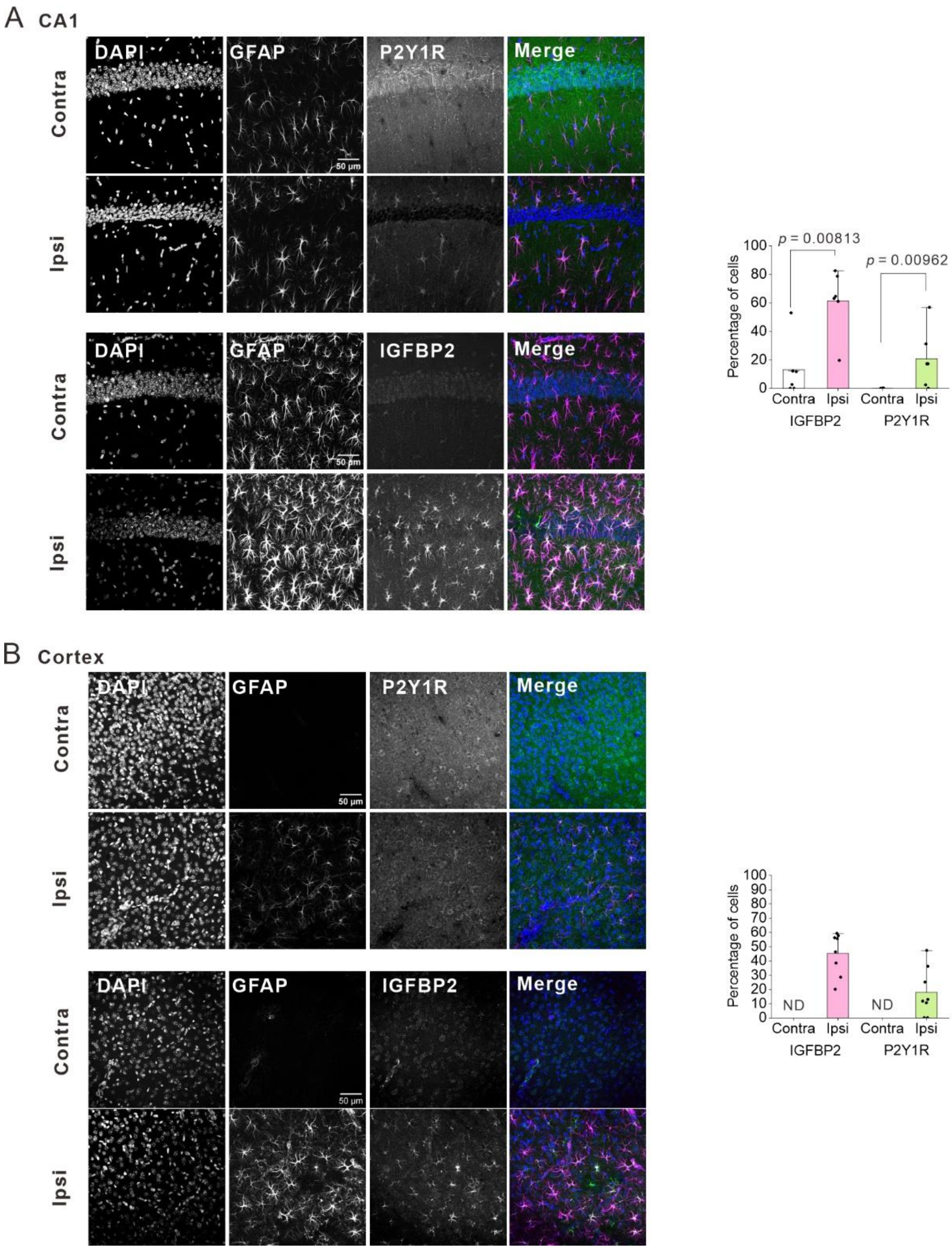
A CA1
DAPI
GFAP
P2Y1R
Merge
Contra
Ipsi
50 μm
DAPI
GFAP
IGFBP2
Merge
Contra
Ipsi
50 μm
p = 0.00813
p = 0.00962
Percentage of cells
100
80
60
40
20
0
Contra
Ipsi
Contra
Ipsi
IGFBP2
P2Y1R
B Cortex
DAPI
GFAP
P2Y1R
Merge
Contra
Ipsi
50 μm
DAPI
GFAP
IGFBP2
Merge
Contra
Ipsi
50 μm
Percentage of cells
100
90
80
70
60
50
40
30
20
10
0
ND
ND
Contra
Ipsi
Contra
Ipsi
IGFBP2
P2Y1R

Supplementary Figure 8. IGFBP2 and P2Y1R are co-upregulated in reactive astrocytes in **the cortex and the hippocampus of MCAO.**

Immunohistochemistry for IGFBP2 and P2Y1R in reactive astrocytes in MCAO model for hippocampal CA1 region (A) and the cortex (B). The bar graph shows a summary of P2Y1R- and IGFBP2-positive GFAP-positive astrocytes (n=6-8 slices from 4 mice, two-sided Mann-Whitney U test). ND, the percentage of cells was not determined because of the low level of GFAP expression in the contralateral side of the cortex. Data are presented as mean ± s.d. Source data are provided as a Source Data file. Source data are provided as a Source Data file.

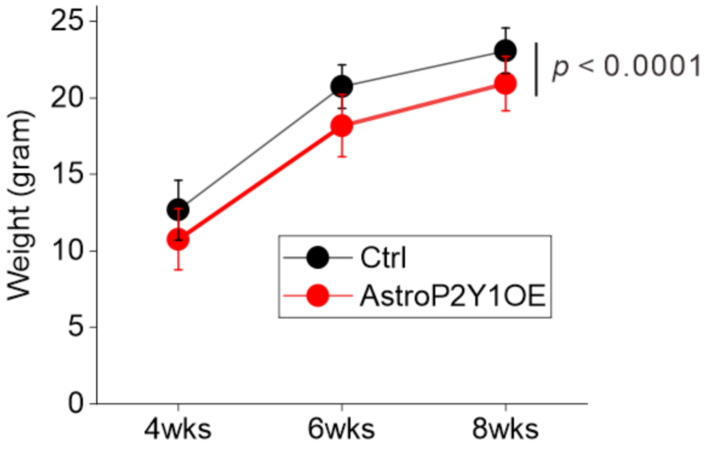

Supplementary Figure 9. Developmental changes in weight of AstroP2Y1OE mice.
Body weight of P2Y1R transgenic mice. Control, N=16; AstroP2Y1OE, N=11 (two-way repeated measures ANOVA and Tukey's test). Data are presented as mean ± s.e.m. Black, control; red, AstroP2Y1OE. Source data are provided as a Source Data file.
