# Supplementary material for: Disease-relevant upregulation of P2Y1 receptor in astrocytes enhances neuronal excitability via IGFBP2
Source: Nat Commun. 2024 Aug 8;15:6525. doi: 10.1038/s41467-024-50190-7 (PMC11310333; doi:10.1038/s41467-024-50190-7)
Supplement: Supplementary file 3 — Description of Additional Supplementary Files [file 41467_2024_50190_MOESM3_ESM.docx]

**Description of Additional Supplementary Files**

**Disease-relevant upregulation of P2Y1 receptor in astrocytes enhances neuronal excitability via IGFBP2**

Eiji Shigetomi^1,2^*^,Ψ^, Hideaki Suzuki^1,2,^*, Yukiho J. Hirayama^1^, Fumikazu Sano^1,2,3^, Yuki Nagai^1,2^, Kohei Yoshihara^4^, Keisuke Koga^4,5^, Toru Tateoka^6^, Hideyuki Yoshioka^6^, Youichi Shinozaki^1,2^, Hiroyuki Kinouchi^6^, Kenji F. Tanaka^7^, Haruhiko Bito^8^, Makoto Tsuda^4,9^ & Schuichi Koizumi^1,2,Ψ^

^1^Department of Neuropharmacology, Interdisciplinary Graduate School of Medicine, University of Yamanashi, Yamanashi 409-3898, Japan.

^2^Yamanashi GLIA center, University of Yamanashi, Yamanashi 409-3898, Japan.

^3^Department of Pediatrics, Faculty of Medicine, University of Yamanashi, Yamanashi 409-3898, Japan.

^4^Department of Molecular and System Pharmacology, Graduate School of Pharmaceutical Sciences, Kyushu University, Fukuoka 812-8582, Japan.

^5^Department of Neurophysiology, Hyogo College of Medicine, Hyogo 663-8501, Japan.

^6^Department of Neurosurgery, Interdisciplinary Graduate School of Medicine, University of Yamanashi, Yamanashi 409-3898, Japan.

^7^Division of Brain Sciences, Institute for Advanced Medical Research, Keio University School of Medicine, Tokyo 160-8582, Japan.

^8^Department of Neurochemistry, Graduate School of Medicine, The University of Tokyo, Tokyo 113-0033, Japan.

^9^Department of Life Innovation, Graduate School of Pharmaceutical Sciences, Kyushu University, Fukuoka 812-8582, Japan.

^Ψ^Corresponding author. Eiji Shigetomi, [eshigetomi@yamanashi.ac.jp](mailto:eshigetomi@yamanashi.ac.jp); Schuichi Koizumi, [skoizumi@yamanashi.ac.jp](mailto:skoizumi@yamanashi.ac.jp)

**This file includes:**

Supplementary Videos 1 to 3

Supplementary Data 1

Supplementary Video 1.

Description: Neuronal Ca^2+^ imaging using jRGECO1a in the stratum radiatum of the CA1 region in a hippocampal slice.

Closed circles appear during the EFS of the Schaffer collaterals.

Supplementary Video 2.

Description: Astrocytic Ca^2+^ imaging using GCaMP6f in the stratum radiatum of the CA1 region in a hippocampal slice.

Closed circles appear during the EFS of the Schaffer collaterals.

Supplementary Video 3.

Description: Merge of Videos 1 and 2.

Magenta and green colors indicate jRGECO1a and GCaMP6f, respectively.

Supplementary Data 1.

Description: Data Reporting FPKM values for genes of hippocampal astrocyte RNA-seq.
