# Supplementary material for: Disease-relevant upregulation of P2Y1 receptor in astrocytes enhances neuronal excitability via IGFBP2
Source: Nat Commun. 2024 Aug 8;15:6525. doi: 10.1038/s41467-024-50190-7 (PMC11310333; doi:10.1038/s41467-024-50190-7)
Supplement: Supplementary file 8 — Reporting Summary [file 41467_2024_50190_MOESM8_ESM.pdf]

nature portfolio

Corresponding author(s): Eiji Shigetomi/Schuichi Koizumi

Last updated by author(s): May 14, 2024

Reporting Summary

Nature Portfolio wishes to improve the reproducibility of the work that we publish. This form provides structure for consistency and transparency in reporting. For further information on Nature Portfolio policies, see our Editorial Policies and the Editorial Policy Checklist.

Statistics

For all statistical analyses, confirm that the following items are present in the figure legend, table legend, main text, or Methods section.

n/a	Confirmed	
☐	☒	The exact sample size (*n*) for each experimental group/condition, given as a discrete number and unit of measurement
☒	☐	A statement on whether measurements were taken from distinct samples or whether the same sample was measured repeatedly
☐	☒	The statistical test(s) used AND whether they are one- or two-sided<br>*Only common tests should be described solely by name; describe more complex techniques in the Methods section.*
☒	☐	A description of all covariates tested
☒	☐	A description of any assumptions or corrections, such as tests of normality and adjustment for multiple comparisons
☐	☒	A full description of the statistical parameters including central tendency (e.g. means) or other basic estimates (e.g. regression coefficient) AND variation (e.g. standard deviation) or associated estimates of uncertainty (e.g. confidence intervals)
☐	☒	For null hypothesis testing, the test statistic (e.g. *F*, *t*, *r*) with confidence intervals, effect sizes, degrees of freedom and *P* value noted<br>*Give P values as exact values whenever suitable.*
☒	☐	For Bayesian analysis, information on the choice of priors and Markov chain Monte Carlo settings
☒	☐	For hierarchical and complex designs, identification of the appropriate level for tests and full reporting of outcomes
☒	☐	Estimates of effect sizes (e.g. Cohen's *d*, Pearson's *r*), indicating how they were calculated

Our web collection on statistics for biologists contains articles on many of the points above.

Software and code

Policy information about availability of computer code

Data collection	Imaging data for genetically encoded Ca2+ indicators, iGluSnFR, GRABATP1.0 were conducted on Fluoview FVMPE-RS two-photon laser scanning microscope using Fluoview FV31S-SW software (Olympus).<br>Imaging data for IHC was conducted with Fluoview FV1200 microscope using Fluoview (ver4.2c) software (Olympus).<br>RNA-seq was performed on DNBseq PE100 (MGI). Reads were aligned to the mouse mm10 reference genome using IGV (Broad Institute/ UCSD).<br>EEG recordings were performed using a PowerLab 26T (ADInstruments).<br>Electrophysiological recordings were performed using MultiClamp 700B controlled by pCLAMP10.5 or pCLAMP10.3 (Axon Instruments).
Data analysis	Imaging data were analyzed on FIJI (ImageJ 1.54f).<br>RNA-seq data were analyzed Dr. Tom software (BGI).<br>EEG recordings and analyzed using LabChart 8 software (ADInstruments).<br>Electrophysiological recordings were analyzed by Clampfit10.5 (Axon Instruments).<br>Imaging, RNA-seq, EEG, behavior, and electrophysiology data were plotted and statistical analysis was performed with OriginPro 2022 SR1 (9.9.0.225)

For manuscripts utilizing custom algorithms or software that are central to the research but not yet described in published literature, software must be made available to editors and reviewers. We strongly encourage code deposition in a community repository (e.g. GitHub). See the Nature Portfolio guidelines for submitting code & software for further information.

Data

Policy information about availability of data

All manuscripts must include a data availability statement. This statement should provide the following information, where applicable:

- Accession codes, unique identifiers, or web links for publicly available datasets
- A description of any restrictions on data availability
- For clinical datasets or third party data, please ensure that the statement adheres to our policy

Source data that supports all findings in this study are available as a supplemental table and source data. Due to their large size, imaging datasets are available upon request. The RNA-seq data are available at GEO with accession ID GSE242450. The analyzed RNA-seq data are provide as Supplementary data Excel file.

Research involving human participants, their data, or biological material

Policy information about studies with human participants or human data. See also policy information about sex, gender (identity/presentation), and sexual orientation and race, ethnicity and racism.

Reporting on sex and gender	NA
Reporting on race, ethnicity, or other socially relevant groupings	NA
Population characteristics	NA
Recruitment	NA
Ethics oversight	NA

Note that full information on the approval of the study protocol must also be provided in the manuscript.

Field-specific reporting

Please select the one below that is the best fit for your research. If you are not sure, read the appropriate sections before making your selection.

☒ Life sciences ☐ Behavioural & social sciences ☐ Ecological, evolutionary & environmental sciences

For a reference copy of the document with all sections, see nature.com/documents/nr-reporting-summary-flat.pdf

Life sciences study design

All studies must disclose on these points even when the disclosure is negative.

Sample size	No statistical methods were used to pre-determine the sample size, but our sample sizes were consistent with those of similar studies (including Haustein et al 2014; Chai et al 2017; Saito et al 2018).
Data exclusions	No data were excluded.
Replication	All experimental findings were reproduced in multiple independent experiments. The number of biological replicates for each experiment is indicated in the figure legends. Behavioral experiments were done in 2-4 batches from the breeding colony.
Randomization	Mice were randomly assigned to experiments. Only male mice were used.
Blinding	For behavioral analysis and EEG recordings, the experimenter was blinded during data collection. For other ex vivo experiments, the experimenters were not blinded during data collection. Blinding was not possible for those ex vivo experiments because the experimenter could distinguish genotype by observing the brain structure (smaller hippocampi and enlarged ventricles).

Reporting for specific materials, systems and methods

We require information from authors about some types of materials, experimental systems and methods used in many studies. Here, indicate whether each material, system or method listed is relevant to your study. If you are not sure if a list item applies to your research, read the appropriate section before selecting a response.

Materials & experimental systems

n/a	Involved in the study
	[x] Antibodies
	[x] Eukaryotic cell lines
[x]	Palaeontology and archaeology
	[x] Animals and other organisms
[x]	Clinical data
[x]	Dual use research of concern
[x]	Plants

Methods

n/a	Involved in the study
[x]	ChIP-seq
[x]	Flow cytometry
[x]	MRI-based neuroimaging

Antibodies

Antibodies used

Primary antibodies:
Rabbit anti-IGFBP2 antibody, Abcam, Cat# ab188200, RRID:AB_2938998
Mouse anti-IGFBP2 antibody, Santa Cruz Biotechnology, Cat# sc-515134
Rat anti-GFAP antibody, Thermo Fisher Scientific; RRID: AB_2532994
Rabbit anti-P2Y1R antibody, Alomone Labs; RRID: AB_2040070
Rabbit anti-SOX9 antibody, Millipore; RRID: AB_2239761
Rat anti-mCherry (16D7), Thermo Fisher Scientific, RRID: AB_2536611
Mouse anti-hemagglutinin (HA) antibody (1:1,000, BioLegend, RRID: AB_2565006
Guinea pig anti-GLT-1 antibody, Sigma-Aldrich: RRID:AB_90949
Rabbit anti-GLAST antibody, Nittobo Medical, Cat# MSFR102120

Secondary antibodies:
Anti-mouse IgG Alexa Fluor 488, RRID: AB_2536161
Anti-rabbit IgG Alexa Fluor 405, RRID: AB_221605
Anti-rabbit IgG Alexa Fluor 488, RRID: AB_143165
Anti-rabbit IgG Alexa Fluor 546, RRID: AB_2534093
Anti-rat IgG Alexa Fluor 488, RRID: AB_2534125
Anti-rat IgG Alexa Fluor 546, RRID: AB_2534125
Anti-guinea pig IgG Alexa Fluor 488, RRID:AB_2534117

Validation

Antibodies were chosen on a literature review. Validation was determined by reviewing the manufacture's literature, other published studies, and our prior studies (Shinozaki et al 2017; Shigetomi et al 2018; Saito et al 2018).

Rabbit anti-IGFBP2 antibody and mouse anti-IGFBP2 antibody were validated by the suppliers.

Rabbit anti-IGFBP2 antibody was validated by Abcam by western blot and immunofluorescence.
Mouse anti-IGFBP2 antibody was validated by Santa Cruz Biotechnology by western blot.

Eukaryotic cell lines

Policy information about cell lines and Sex and Gender in Research

Cell line source(s)	Adeno-associated viruses were produced in HEK293 cells, obtained from Agilent.
Authentication	No
Mycoplasma contamination	Not tested
Commonly misidentified lines (See ICLAC register)	NA

Animals and other research organisms

Policy information about studies involving animals; ARRIVE guidelines recommended for reporting animal research, and Sex and Gender in Research

Laboratory animals	C57BL6/J mice, Mlc1-tTA BAC transgenic mice, and P2ry1tetO knockin mice were used in this study. All the mice were adult (7-11 weeks) at time of surgery, behavioral experiments, electrophysiological recordings, or isolation of RNAs. C57BL/6J mice were purchased from Japan SLC. Mice were housed on a 12-hour light (6 a.m.)/dark (6 p.m.) cycle with ad libitum access to water and rodent chow.
Wild animals	None

Reporting on sex	All mice used in this study were male.
Field-collected samples	None
Ethics oversight	The protocol was approved by the Animal Care Committee of Yamanashi University (Chuo, Yamanashi, Japan; Approval No. A29-7).

Note that full information on the approval of the study protocol must also be provided in the manuscript.
